# Malnutrition as predictor of poor outcome after total hip arthroplasty

**DOI:** 10.1007/s00264-020-04892-4

**Published:** 2020-11-26

**Authors:** Sandra Eminovic, Gabor Vincze, Doris Eglseer, Regina Riedl, Patrick Sadoghi, Andreas Leithner, Gerwin A. Bernhardt

**Affiliations:** 1 Department of Orthopedics and Traumatology, Medical University of Graz, Auenbruggerplatz 5, 8036 Graz, Austria; 2 Institute of Nursing Science, Medical University of Graz, Universitätsplatz 4, 8010 Graz, Austria; 3 Institute for Medical Informatics, Statistics and Documentation, Medical University of Graz, Auenbruggerplatz 2, 8036 Graz, Austria

**Keywords:** Malnutrition, PEM, Surgery, Total hip arthroplasty, Post-operative outcome

## Abstract

**Introduction:**

The aim of this study was to assess the prevalence of protein energy malnutrition (PEM) and correlation with poor post-operative outcome in the elderly undergoing primary total hip arthroplasty (THA).

**Hypothesis:**

Patients with PEM would have inferior post-operative outcome after THA.

**Materials and method:**

We retrospectively evaluated the nutritional status of 220 hospitalized patients undergoing THA, 65 years and older. PEM was assessed using serum albumin and total lymphocyte count (TLC). Studied outcome parameters were length of pre-operative and post-operative stay, complications up to six months after surgery and 12-month mortality. Clinical and demographic data were retrieved from medical records from the hospital database.

**Results:**

The prevalence of PEM among patients undergoing THA was 12.3% (27/220). Patients with PEM were significantly older (mean age 81.3 ± 7.0, *p* < 0.001), had a lower BMI (24.7 ± 4.1 kg/m 2, *p* = 0.022), and showed more comorbid conditions (mean CCI 2.8 ± 2.0, *p* = 0.002) compared with well-nourished patients (age 75.6 ± 6.2, BMI 26.8 ± 4.3 kg/m 2, CCI: 1.7 ± 1.7). Length of pre-operative stay differed significantly (*p* < 0.001) between PEM (median 7, range 1–36 days) and non PEM (median 1, range 1–22 days). In the PEM group, 12 (44.4%) patients had post-operative complications within six months after OP and 15 (7.8%) patients in the non PEM group (HR = 6.3, 95% CI 1.7–23.1).

**Conclusion:**

We observed a higher post-operative complication rate for malnourished patients undergoing elective THA. These results underline the importance of pre-operative nutritional assessment in the elderly. Therefore, serum albumin and TLC are valuable clinical markers of PEM and the post-operative outcome.

## Introduction

Osteoarthritis (OA) is one of the most common joint disorders in the elderly with a prevalence up to 45% in general population [1]. To alleviate the consequences of OA as pain or disability, total joint replacement (TJR) is commonly performed. One of the most often affected joints with OA is the hip requiring total hip arthroplasty (THA) in end stage osteoarthritis.

Besides, elderly patients show a higher risk for malnutrition. For those affected, this can lead to longer hospital stays with increased morbidity and higher mortality [2, 3]. Poor nutritional status tends to result in impaired wound healing or an increased risk of infections [4].

Successful rehabilitation after THA is determined by patients’ peri-operative health status. Hospitalization represents a stressful event, and consequently, the nutritional status tends to deteriorate during the hospital stay. Several studies are demonstrating the association of poor nutritional status and adverse outcomes in surgical patients [2, 5].

Definitive prevalence of malnutrition seems to be unclear, as there is no consensus on the best screening method. Various combinations of indicators have led to a range of scales. Consequently, the reported prevalence in patients undergoing elective joint arthroplasty varies widely between 8.5 and 30% [6]. Identifying the risk of adverse post-operative outcomes laboratory values may be used as a nutritional screening tool for hospitalized persons [7]. To assess protein energy malnutrition (PEM), biochemical parameters such as serum albumin and total lymphocyte count (TLC) as predictors of outcome in hip fracture patients have been used in literature [2, 3]. Malnutrition seems to be a common causative factor for hip fractures [8] as several studies demonstrated poor outcome in malnourished hip fracture patients [5, 9]. So far only a few studies indicated post-operative outcome in patients undergoing elective arthroplasty [6]. Given that total arthroplasty among older people is associated with high post-operative complications, the pre-operative nutritional status can be emphasized for patients’ recovery [2, 5]. However, the controversial impact of pre-operative nutritional status in patients undergoing THA has not fully been evaluated in the literature.

The aim of this study was therefore to assess the prevalence of PEM and to assess whether pre-operative nutritional parameters are associated with poor post-operative outcome in the elderly undergoing primary total hip arthroplasty. Our hypothesis was that patients with PEM would have inferior post-operative outcome after THA.

## Materials and methods

All patients who underwent primary THA at a department of orthopaedic surgery in an 8-year period were retrospectively studied. Patients´ clinical and demographic data were retrieved from medical records from the hospital database. Patients’ chronic medical conditions were assessed with the Charlson comorbidity index (CCI) [10] based on the medical history. All 19 comorbidities necessary to calculate the CCI and the age of the patients were taken into account. To assess patients’ pre-operative physical status, American Society of Anesthesiologists (ASA) rating of operative risk was used from medical records. Patients suitable for operation were classified as ASA categories I, II, III, and IV as used previously [11].

Serum albumin and TLC were retrieved from a computerized laboratory database. All testing was performed by the same laboratory with standardized reference values for each test. All samples were taken at admission and on the first post-operative day. Pre-operative values of <3.5 g/dl of serum albumin and < 1.5 g/l of TLC were considered as PEM [3]. Given that OA affects mostly older individuals we decided to include patients 65 years and older. Patients without laboratory tests performed pre-operatively were excluded. Pre-operative transfusion rates were not evaluated, and we do not pre-operatively assess osteoporosis through DEXA. The included patients were divided into two groups:

- Non PEM: pre-operative albumin ≥ 3.5 g/dl or TLC ≥ 1.5 g/l
- PEM: pre-operative albumin < 3.5 g/dl and TLC < 1.5 g/l

Time from admission to operation (pre-operative stay), length of post-operative stay, post-operative complications after three and six months, and 12-month post-operative mortality were assessed and compared between the groups. Post-operative complications were defined as any deviation of a routine post-operative hospital stay. They were classified dichotomously (present/absent) and represented at least one of the frequent short-term complications including surgical and non-surgical complications such as infections, pulmonary intricacies, impaired wound healing, or vascular complications. Post-operative complications included subsequent or repeated re-admissions that followed the initial hospitalization and could not be foreseen at the time of discharge. Also included were acute admissions to other wards while patients were hospitalized at our department.

## Statistical analysis

Continuous variables are presented as mean ± standard deviation or median and minimum, maximum, and categorical variables as frequencies and percentages. Group comparisons were performed by using Chi-square or Fishers exact test and *t* test or Mann-Whitney U test, as appropriate. For complications six months after surgery, Kaplan-Meier curves are presented and hazard ratios (HR) with their corresponding 95% confidence intervals (CI) were estimated using a Cox proportional hazard models including group (PEM vs. non PEM), gender, age, type of diagnosis, time to operation, pre-operative C-reactive protein (CRP), and pre-operative hemoglobin (HB). A *p* value of < 0.05 was considered to indicate statistical significance. All *p* values are regarded in an explorative sense. The statistical analysis was performed using the statistical software SPSS, Version 22 (SPSS Inc., Chicago, Ill).

## Results

### Sample characteristics

A total of 1183 patients underwent primary THA during the study period. Pre-operative serum albumin and TLC levels were available for 220 (18.6%) patients. The majority of the patients were female (*n* = 144, 65.5%) and the mean age was 76.3 ± 6.6 years, ranging from 65 to 95 years (Table 1). The median time of pre-operative stay was 2 (1–36) days and median time of post-operative stay was 14 (1–50) days. Twenty-seven (12.3%) patients had post-operative complications or were readmitted to hospital within six months. Three (1.4%) patients died within 12 months after their operation.

**Table 1:** Demographic data and patient characteristics

Characteristics	Study group *n* = 220
Gender	
Female	144 (65.5%)
Male	76 (34.5%)
Age (years)	76.3 ± 6.6
Diagnosis	
OA	195 (88.6%)
Hip fracture	25 (11.4%)
BMI (kg/m^2^), *n* = 185	26.5 ± 4.3
CCI	1.8 ± 1.8
ASA I	2 (0.9%)
ASA II	44 (20%)
ASA III	105 (47.7%)
ASA IV	52 (23.6%)

OA = Osteoarthritis

CCI = Charlson Comorbidity Index

ASA = American Society of Anesthesiologists

### Prevalence of PEM

The prevalence of PEM in the patient cohort was 12.3% (*n* = 27). Pre-operative albumin levels < 3.5 g/dl and TLC ≥ 1.5 g/l were observed in 7 (3.2%) patients, and pre-operative albumin levels ≥ 3.5 g/dl and TLC < 1.5 g/l were observed in 108 (49.1%) patients.

### Clinical characteristics of patients with PEM and without PEM

Patients with PEM were significantly older (mean age 81.3 ± 7.0, *p* < 0.001), had a lower BMI (24.7 kg/m^2^ ± 4.1 kg/m^2^, *p* = 0.022) and showed more comorbid conditions (mean CCI 2.8 ± 2.0, *p* = 0.002) compared to patients without PEM (mean age 75.6 ± 6.2, mean BMI 26.8 ± 4.3 kg/m^2^, mean CCI: 1.7 ± 1.7). The median pre-operative stay in the PEM group was 7 (1–36) days vs. 1 (1–22) day in the non PEM group (*p* < 0.001). No differences were found in median length of post-operative stay between the groups (*p* < 0.544). Three patients from the PEM group died within 12 months after surgery. Overall characteristics are presented in Table 2.

**Table 2:** Clinical characteristics according to PEM groups

Variable	Non-PEM *n* = 193	PEM *n* = 27	*p* value
Age (years)	75.6 ± 6.2	81.3 ± 7.0	< 0.001
Gender			0.390
Female	124 (64.2%)	20 (74.1%)	
Male	69 (35.8%)	7 (25.9%)	
BMI	26.8 (± 4.3)	24.7 (± 4.1)	0.022
CCI	1.7 (± 1.7)	2.8 (± 2.0)	0.002
Diagnosis			
OA	178 (92.2%)	17 (63.0%)	0.001
Hip fracture	15 (7.8%)	10 (37.0%)	
ASA			0.001
Grade I	2 (100%)	0	
Grade II	44 (24.9%)	0	
Grade III	93 (52.5%)	12 (46.2%)	
Grade IV	38 (21.5%)	14 (53.8%)	
Pre-operative stay (days)	1 (1–22)	7 (1–36)	< 0.001
Length of post-operative stay (days)	14 (4–36)	14 (1–50)	< 0.544

ASA = American Society of Anesthesiologists

OA = Osteoarthritis

### Association between PEM and post-operative complications

In the PEM group (40.7%; *n* = 11) significantly (*p* < 0.001) more patients had post-operative complications within three months compared with the non PEM group (6.2%; *n* = 12). The morbidity rate within 6 months after surgery was 44.4% (*n* = 12) in patients with PEM and 7.8% (*n* = 15) in patients without PEM (*p* < 0.001). Complications as causes for acute admission or re-admission in 220 patients are summarized in Table 3.

**Table 3:** Complications causing acute admissions or re-admissions within 6 months after surgery

Post-operative complications	Non-PEM (*n* = 193)	PEM (*n* = 27)
Urinary complications	3 (1.6%)	2 (7.4%)
Pulmonary complications	1 (0.5%)	5 (18.5%)
Wound complications	2 (1%)	2 (7.4%)
Periprosthetic fractures	2 (1%)	0
Post-operative swelling	2 (1%)	0
Vascular complications	1 (0.5%)	1 (3.7%)
Septic arthritis of the hip	0	1 (3.7%)
Prosthetic infection	1 (0.5%)	0
Other complications	3 (1.6%)	1 (3.7%)
Total	15 (7.8%)	12 (44.4%)

**Fig. 1:**
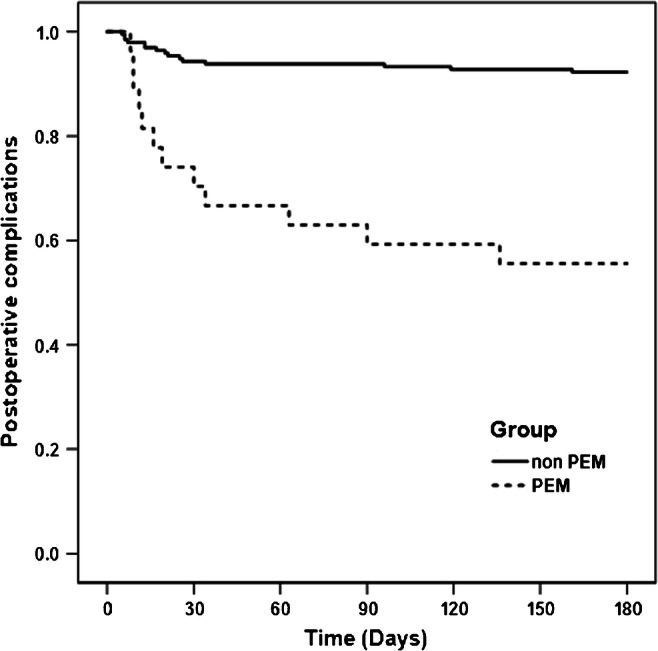
Kaplan-Meier curves for post-operative morbidity between protein energy malnutrition (PEM) and no- protein energy malnutrition (non PEM) groups

In multivariate analysis, an increased risk for six month post-operative morbidity was observed for patients with PEM compared with the non PEM group (HR 6.3, 95% CI: 1.7–23.1), adjusted for gender, age, type of diagnosis, time to operation, CRP, and HB. The Kaplan-Meier curves for post-operative morbidity between groups are shown in Fig. 1.

## Discussion

PEM at hospital admission was present among 12.3% of patients. The aim of this retrospective study was to assess the prevalence of PEM and to investigate whether pre-operative nutritional parameters are associated with poor post-operative outcome in elderly patients undergoing primary total hip arthroplasty.

We could prove our hypothesis aiming that patients with PEM have inferior post-operative outcome after THA. We observed a higher risk for six month post-operative complications in patients with PEM compared with patients without PEM (HR 6.3, 95% CI: 1.7–23.1). Furthermore, patients with PEM were significantly older, had a lower body mass index, and showed more comorbid conditions. Malnourished patients had a higher length of pre-operative stay. However, for the length of post-operative stay, no differences were observed between the groups.

The evaluated PEM prevalence (12.3%) is not consistent with that reported by a related study [12]. Assessing PEM with same haematological markers of serum albumin and TLC, Nicholson et al. [12] investigated a rate of 30% in elective patients based on a smaller study population (*n* = 90) compared with our sample (*n* = 220). Our findings are similar to that of Huang et al. [6] where a prevalence of 8.5% was observed in patients undergoing elective joint arthroplasty. Investigations by Nicholson et al. [13] demonstrated a high rate (60%) of malnutrition in hip fracture patients which is in accordance with our evaluation. PEM was observed in 8.7% (17/195) of patients with OA but 40% (10/25) in hip fracture patients undergoing hip arthroplasty. In our study PEM on admission seems to be quite prevalent not only in older patients but also in hip fracture patients. This finding is supported by other clinical trials in orthopaedic patients [6]. Moreover several studies described negative consequences of malnutrition on mortality, morbidity, quality of life, or healthcare costs [2, 12, 14]. Recently, Lee et al. [2] identified low pre-operative albumin levels and hip surgery as negative predictors for home return in patients over the age of 85.

However, a limitation of this study is that only a small number of patients had both albumin levels and TLC taken at admission. Out of 1183 initially screened operated patients, in 220 (18.6%) both parameters were available. Serum albumin was not routinely collected at our department. Usually pre-operative laboratory records were determined a few weeks earlier through general practitioners or other institutions.

In this study we used biochemical nutritional markers to evaluate nutritional status as recommended to investigate PEM [3]. Because of the retrospective nature of the study we could not include more accepted and powerful tests such as Mini Nutritional Assessment (MNA) or Malnutrition Universal Screening Tool (MUST). According to other studies, the post-operative use of biochemical parameters is not recommended for clinical practice. Biochemical parameters are not reliable due to the fast decrease after acute illness or trauma [15]. We found that post-operatively low serum albumin and TLC occurred in 69.5% (146/210) of the patients regardless of their nutritional status at admission. The use of serum albumin as single variable evaluating PEM is insufficient. Using only one nutritional parameter could underestimate the prevalence of malnutrition in hospitalized patients [16]. However, our study used more than one nutritional parameter as indicator of malnutrition [3]. We additionally analyzed albumin and TLC values separately. Low albumin levels and normal TLC ≥ 1.5 g/l were recorded for 7 (3.2%) patients and a low TLC levels and normal albumin ≥ 3.5 g/dl for 108 (49.1%) patients.

Several studies demonstrated positive aspects of fast-track arthroplasty and indicated the importance of patient characteristics that could influence successful rehabilitation after fast-track total hip or total knee arthroplasty [17]. In this context the nutritional status seems to play an important factor. Rudasill et al. created a pre-operative risk model to predict hypoalbuminemia at the patients undergoing THA. Individuals with three or more risk factors in the 7-point model were predicted to have hypoalbuminemia in 20.4% of the cases [18]. Consequently malnutrition at admission could represent a relevant exclusion criterion to perform fast-track surgery in affected patients. We have shown that basic laboratory values can predict risk for poor post-operative outcome in patients for elective surgery. Our results are comparable to that of Lu et al. [8] who examined a poor post-operative outcome in patients after a hip fracture surgery. Malnourished patients had an increased length of stay and showed a higher one year mortality rate compared with well-nourished patients. The results of the meta-analysis of seven cohort studies published by Tsantes et al. showed also that malnutrition is associated with higher infection rates after THA [19].

The used nutritional parameters seem to be an inexpensive method identifying surgical patients early at risk for poor outcome. Our findings highlight the importance of punctual nutritional assessment to detect dietary deficits. A simple pre-operative assessment can additionally identify patients at risk. As a consequence, adequate dietary supplements can be provided and economic burden of readmissions may be avoided [20]. This is necessary, especially since low intake of calories and proteins, seems to be associated with low muscle mass which may result in sarcopenia. A Cochrane review of 41 studies showed that oral nutritional supplementation started before or soon after surgery can decrease complication rates in the first 12 months after hip fracture [21]. Vetrano et al. [22] underlines the need for nutritional support as muscle wasting can be decreased. Sarcopenia represents an important risk factor for adverse outcomes in the elderly [2, 20, 23] like in our study. Sarcopenia is a screenable and optimizable risk factor, which needs further research. Our results showed that either normal weight or overweight does not exclude malnutrition, showing a mean BMI in the PEM group of 24.7 ± 4.1 kg/m^2^. Babu et al. evaluated the impact of the psoas-lumbar vertebral index of patients undergoing THA to be a risk factor for PJI [24]. Ryniecki et al. found the same results at revisional THA showing that patients having pre-operative hypoalbuminemia had an increased risk facing peri-operative complications, compared with those without hypoalbuminemia [25].

## Conclusion

We observed in a large cohort a higher post-operative complication rate for malnourished patients undergoing elective THA compared with well-nourished patients. These results underline the importance of pre-operative nutritional assessment in older surgical patients. Serum albumin and TLC are inexpensive and valuable clinical markers of PEM with accurate prognostic evidence of post-operative outcomes. Further prospectively planned studies need to evaluate the impact of PEM and clinical outcome parameters together with the MNA or MUST. Clear guidelines need to be developed to determine cheap markers as a basis for pre-operative preventive treatment with oral supplements to decrease the incidence of post-operative complications.

## Abbreviations and acronyms

PEM: Protein energy malnutrition
THA: Total hip arthroplasty
TLC: Total lymphocyte count
